# Natural human knockouts and Mendelian disorders: deep phenotyping in Italian isolates

**DOI:** 10.1038/s41431-021-00850-9

**Published:** 2021-03-16

**Authors:** Beatrice Spedicati, Massimiliano Cocca, Roberto Palmisano, Flavio Faletra, Caterina Barbieri, Margherita Francescatto, Massimo Mezzavilla, Anna Morgan, Giulia Pelliccione, Paolo Gasparini, Giorgia Girotto

**Affiliations:** 1 Department of Medicine, Surgery and Health Sciences, University of Trieste, Trieste, Italy; 2 Institute for Maternal and Child Health – I.R.C.C.S. “Burlo Garofolo”, Trieste, Italy; 3 Division of Genetics and Cell Biology, San Raffaele Scientific Institute, Milan, Italy

**Keywords:** Genetics research, DNA sequencing, Rare variants

## Abstract

Whole genome sequencing (WGS) allows the identification of human knockouts (HKOs), individuals in whom loss of function (LoF) variants disrupt both alleles of a given gene. HKOs are a valuable model for understanding the consequences of genes function loss. Naturally occurring biallelic LoF variants tend to be significantly enriched in “genetic isolates,” making these populations specifically suited for HKO studies. In this work, a meticulous WGS data analysis combined with an in-depth phenotypic assessment of 947 individuals from three Italian genetic isolates led to the identification of ten biallelic LoF variants in ten OMIM genes associated with known autosomal recessive diseases. Notably, only a minority of the identified HKOs (*C7*, *F12*, and *GPR68* genes) displayed the expected phenotype. For most of the genes, instead, (*ACADSB*, *FANCL*, *GRK1*, *LGI4*, *MPO*, *PGAM2*, and *RP1L1*), the carriers showed none or few of the signs and symptoms typically associated with the related diseases. Of particular interest is a case presenting with a *FANCL* biallelic LoF variant and a positive diepoxybutane test but lacking a full Fanconi anemia phenotypic spectrum. Identifying KO subjects displaying expected phenotypes suggests that the lack of correct genetic diagnoses may lead to inappropriate and delayed treatment. In contrast, the presence of HKOs with phenotypes deviating from the expected patterns underlines how LoF variants may be responsible for broader phenotypic spectra. Overall, these results highlight the importance of in-depth phenotypical characterization to understand the role of LoF variants and the advantage of studying these variants in genetic isolates.

## Introduction

One of the best ways to investigate the function of a gene consists in studying the phenotypic consequences of a gene knockout event [1], in particular in animal models such as mice and rats, which share with humans approximately 80% of their genome. With the availability of high-throughput sequencing technologies, it has become feasible to sequence the entire genome of thousands of individuals opening new perspectives in the study of the effect of genes knockout events directly in humans [2]. Large-scale genome sequencing allowed the identification of loss of function (LoF) variants that include splice acceptor, splice donor, stop gained, stop lost, start lost, frameshift, transcript ablation, and transcript amplification variants. Individuals who carry biallelic LoF variants may be defined as human knockouts (HKOs) [3]. These LoF events may occur in genes already known to be implicated in severe genetic diseases or involve novel genes; such variants may be related to an extensive range of phenotypes, from disease-causing variants to variants responsible for the common inter-individual variability and even to variants that are beneficial to the carrier [4]. Recent studies have highlighted how each healthy subject may carry up to 100 LoF variants in his/her genome, most of them heterozygous, and thus presenting with 20 completely inactivated genes, some associated with Mendelian disorders and some other in “non-essential” genes. The absence of clinical signs could be explained by the role of modifier genes or by the possible presence in these individuals of other protective genetic pathways [5, 6].

**Fig. 1:**
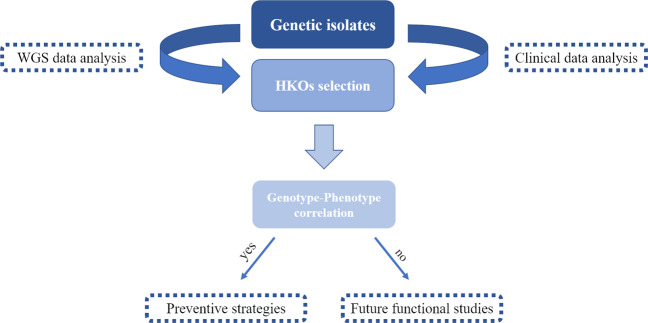
Study workflow. The scheme represents the overall workflow of the study. WGS and clinical data from Italian isolated populations have been combined to select putative HKOs. In case of positive genotype–phenotype correlation, preventive strategies could be applied, whereas whenever the correlation results negative, functional studies are needed to better clarify the role of the identified variant.

An efficient and cutting-edge approach to study HKOs consists in detecting these subjects in genetic isolates, i.e., populations characterized by few founders, small population size, high rate of inbreeding, and low rate of gene flow [7, 8]. These population characteristics lead to decreased genetic variability and can determine an enrichment in homozygous LoF variants, specifically in genes associated with recessive Mendelian genetic disorders [9]. The Italian Network of Genetic Isolates (INGI) includes several Italian isolated populations, characterized by a high endogamy rate and a particular genetic background, as previously demonstrated [10, 11]. Here, we investigated three INGI cohorts: Carlantino (CAR), a small village located in the Puglia region, Val Borbera (VBI), a valley in the Northwest of Italy, and the Friuli-Venezia Giulia (FVG) Genetic Park, which includes six villages in Northeastern Italy. A recent whole genome sequencing (WGS) study [12] provided an extensive characterization of these three isolated populations, describing their genetic features focusing on homozygous LoF variants. Here we describe the results obtained combining WGS data [12] and deep clinical phenotypes in Italian isolates with the main aim of increasing the knowledge on the role of the identified LoF variants in HKOs (Fig. 1).

## Materials and methods

### Ethical statement

All the experiments have been performed following relevant guidelines and regulations. The study was reviewed and approved by the Ethics Committee of the Institute for Maternal and Child Health – I.R.C.C.S. “Burlo Garofolo” of Trieste (Italy) (2007 242/07). The protocol conformed to the tenets of the Declaration of Helsinki.

### Whole genome sequencing data generation

WGS data have been generated and analyzed, as previously reported by Cocca et al. [12]. Briefly, 947 DNA samples were randomly selected from the three cohorts, and WGS at 6–10× coverage was performed. Specifically, 381 individuals were selected from the Friuli-Venezia Giulia cohort, 433 from the Val Borbera cohort, and 133 from the Carlantino one. After extensive quality control, 926 samples were retained, and the generated data were aligned to the GRCh37/hg19 reference sequence. The aligned data were processed using GATK best practice pipelines [13] in order to generate germinal variant calls for both SNPs and INDELs. Functional annotation was performed using the Variant Effect Predictor tool [14]. Variants annotated as protein-truncating were selected as LoF. Specifically, the following categories were considered: frameshift, splice acceptor, splice donor, stop gained, stop lost, start lost, transcript ablation, and transcript amplification variants [3].

The genetic data described in this manuscript have been submitted to the European Variation Archive and are accessible in Variant Call Format at the following link: https://www.ebi.ac.uk/ena/data/view/PRJEB33648.

### Human knockouts: functional selection and bioinformatic filters

The starting point of our work was a list of 506 LoF variants with a Combined Annotation Dependent Depletion score greater or equal to 20 [15] and for which at least one homozygous carrier was detected in our dataset, as described by Cocca et al. [12]. We first selected only variants in genes already known to be associated with Mendelian disorders, as reported in the Online Mendelian Inheritance in Man^®^ (OMIM; https://www.omim.org/) free-access catalog of human genes and genetic disorders (Supplementary Table 1). In order to identify all the subjects with low-frequency biallelic LoF variants, a total allele frequency upper limit of 1% according to gnomAD (https://gnomad.broadinstitute.org/; date of the last update: May 24, 2020) was applied [16]. Furthermore, only variants affecting genes causative of autosomal recessive disorders were retained, resulting in 13 LoF variants in 13 distinct genes. Finally, for each selected variant, we confirmed whether it was a “total” or “partial” LoF, based on the number of the gene transcripts involved (https://www.ensembl.org/index.html). Specifically, each LoF variant has been classified as “total” if it falls on all coding transcript of a gene or as “partial” if it falls only on some coding transcripts. The ratio between the number of coding transcripts for which the variant is a LoF and the total number of coding transcripts of every gene is reported in Table 1.

**Table 1:** Selected loss of function variants.

Gene	Chromosome	HGVS genomic nomenclature	HGVS coding DNA nomenclature	rsID	gnomAD frequency	Total/partial; n° KO coding transcripts/n° coding transcripts	Identified subjects	Age
*C7* (NM_000587.2)	5	NC_000005.9:g.40980013T>C	NM_000587.2:c.2350+2T>C	rs201240159	0.00028	Total: 1/1	Individual_1	55
*F12* (NM_000505.3)	5	NC_000005.9:g.176829461C>T	NM_000505.3:c.1681-1G>A	rs199988476	0.00039	Total: 1/1	Individual_2	79
*GPR68* (NM_003485.3)	14	NC_000014.8:g.91700389C>A	NM_003485.3:c.1006G>T	rs61745752	0.00092	Total: 4/4	Individual_3	68
*ACADSB* (NM_001609.3)	10	NC_000010.10:g.124797364G>A	NM_001609.3:c.303+1G>A	rs147936696	0.00027	Partial: 2/3	Individual_4 Individual_5	82* 86
*FANCL* (NM_018062.3)	2	NC_000002.11:g.58468447A>G	NM_018062.3:c.2T>C	rs761291501	0.00005	Partial: 6/7	Individual_6	74
*GRK1* (NM_002929.2)	13	NC_000013.10:g.114322401G>A	NM_002929.2:c.699+1G>A	rs1191610272	0	Total: 1/1	Individual_7	69*
*LGI4*	19	NC_000019.9:g.35622287del	ENST00000591633.1:c.636del	rs770752678	0.00003	Partial: 1/4	Individual_8	75*
*MPO* (NM_000250.1)	17	NC_000017.10:g.56350831_56350844del	NM_000250.1:c.1552_1565del	rs536522394	0.00078	Total: 3/3	Individual_9	77
*PGAM2* (NM_000290.3)	7	NC_000007.13:g.44104494del	NM_000290.3:c.532del	rs747947171	0.00004	Total: 1/1	Individual_10	84
*RP1L1* (NM_178857.5)	8	NC_000008.10:g.10480385_10480386insA	NM_178857.5:c.326_327insT	rs771427543	0.00143	Total: 1/1	Individual_11	70

Gene: Genes carrying the selected variants. NM_ is referred to the canonical transcript of each gene, when the variant is reported also on the canonical transcript. HGVS genomic nomenclature: variants description according to the Human Genome Variation Society recommendations for linear genomic reference sequence; genomic data are aligned to the GRCh37/hg19 reference sequence. HGVS coding DNA nomenclature: variants description according to the Human Genome Variation Society recommendations for coding DNA reference sequence. rsID: Reference SNP cluster ID; rsIDs are updated to the latest dbSNP build (154). gnomAD frequency: variant frequency reported in gnomAD total allele frequency. Total/partial: each LoF variant has been classified as “Total” if it falls on all coding transcripts of a gene or as “Partial” if it falls only in some coding transcripts; n° KO coding transcripts/n° coding transcripts: number of coding transcripts for which the variant is a LoF over the total number of coding transcripts of each gene. Identified subjects: HKOs identification number. Age: age of identified subjects at follow-up (2019); individuals marked with an asterisk are deceased and age at first examination is reported.

### Variants confirmation

**Fig. 2:**
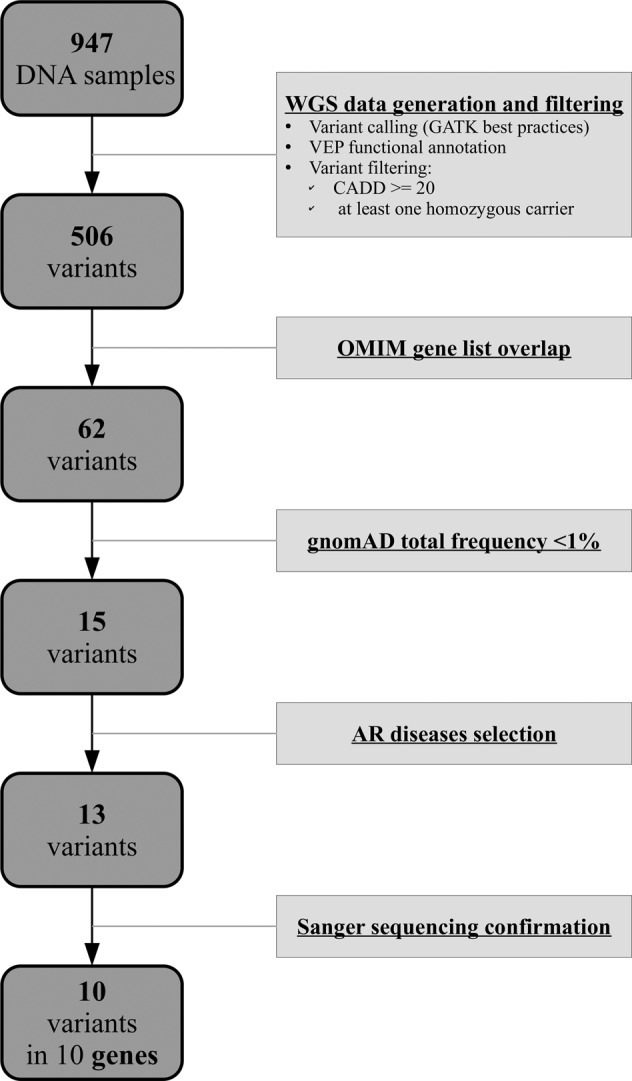
Homozygous loss of function variants selection process. The scheme highlights the variants selection and filtering workflow. WGS data were obtained for 947 samples and processed applying GATK best practice pipelines to generate variant calls for both SNPs and INDELs. After functional annotation with the VEP tool, 506 LoF variants with at least one homozygous carrier and CADD score ≥20 were selected. Further filtering overlapping those variants with OMIM disease-associated genes reduced our target list to 62 variants. A minor allele frequency upper limit of 1% according to data from the gnomAD database (gnomad_AF field) was applied, to retain only low-frequency variants. A final filtering step to select variants in genes causative of autosomal recessive disorders (AR) was performed. The resulting variants underwent confirmation through Sanger Sequencing.

All selected variants underwent Sanger sequencing confirmation. In order to amplify the DNA fragments, a touchdown polymerase chain reaction (PCR) was performed; the success of the PCR reaction was confirmed with electrophoresis and subsequent band visualization through a LED illuminator (FastGene^®^ FAS V; Gel Documentation System). The amplified PCR products were then purified and labeled with BigDye^®^ Terminators (ddNTPs) according to the manufacturer’s protocol. After a second purification step, the DNA fragments were sequenced (Applied Biosystem™ 3500 DX Genetic Analyzer; Thermo Fisher). The filtering and variants confirmation process is summarized in Fig. 2.

### Clinical evaluation and follow-up

The initial clinical evaluation of all the subjects involved in the study comprised the assessment of hundreds of functional parameters, including (1) clinical biochemistry data (over 60 parameters inclusive of a complete blood count with differential, electrolytes, liver enzymes, serum protein, bilirubin, creatinine, insulin and lipase, cholesterol and triglycerides), (2) metabolomics data (obtained through 500 mHz nuclear magnetic resonance spectroscopy serum analysis), (3) bone densitometry, (4) an in-depth sensory evaluation that focused on the analysis of senses (hearing, taste, smell and vision), (5) a cardiovascular, a neurological and an orthodontic evaluation, and (6) a detailed personal and familial history with more than 200 questions asked to each subject. All parameters were systematically collected by professional and trained staff according to a standardized format. Since the parameters collected during the initial sampling were standard for all subjects, in some cases, a clinical follow-up was required in order to gather more details specific for the expected clinical phenotype.

## Results

### Homozygous LoF variants selection and validation

Considering the starting list of 506 LoF variants, only those in genes already known to be associated with autosomal recessive Mendelian disorders were selected (Supplementary Table 1). These variants were filtered as detailed in Materials and methods, obtaining 13 variants in 13 genes. Finally, 10 out of 13 variants were confirmed by Sanger sequencing, and their role was further investigated, looking at the corresponding phenotypes (Table 1). Of note, according to gnomAD, all the selected genes show evidence of LoF tolerance (pLI = 0). However, among them, three (*FANCL*, *PGAM2*, and *RP1L1*) should be more prone to accumulate LoF variants, with an observed vs. expected LoF ratio >1.1. The other seven genes (*C7*, *F12*, *ACADSB*, *GRK1*, *LGI4*, *MPO*, and *GPR68*) are supposedly less prone to accumulate LoF variants, with an observed vs. expected LoF ratio <1.

### Phenotypical characterization of the carriers of the selected loss of function variants

The phenotypes of the subjects carrying homozygous LoF variants have been deeply investigated and compared to the expected ones (Table 2). A brief description of the diseases associated with LoF variants in the selected genes and the relevant clinical findings is reported below.

**Table 2:** OMIM autosomal recessive diseases description and HKOs phenotypical features.

Gene	OMIM disease (MIM number)	Expected phenotypical features	Detected phenotypical features
*C7*	C7 deficiency (#610102)	Increased susceptibility to systemic infections	Meningococcal meningitis, pericarditis, pneumonia, soft tissue infection
*F12*	Factor XII deficiency (#234000)	Prolonged APTT	Prolonged APTT
*GPR68*	Amelogenesis imperfecta, hypomaturation type, IIA6 (#617217)	Enamel abnormalities, multiple caries	Multiples caries and recurrent tooth decay
*ACADSB*	2-methylbutyrylglycinuria (#610006)	Developmental delay and neurological signs	–
*FANCL*	Fanconi anemia, complementation group L (#614083)	Bone marrow failure, skeletal abnormalities, increased cancer risk	Head and neck carcinoma, short stature
*GRK1*	Oguchi disease type 2 (#613411)	Night blindness	–
*LGI4*	Arthrogryposis multiplex congenita, neurogenic, with myelin defect (#617468)	Neurogenic defect with poor or absent myelin formation around peripheral nerves; prenatal onset; usually lethal in utero or in early childhood	–
*MPO*	Myeloperoxidase deficiency (#254600)	Candidiasis	–
*PGAM2*	Glycogen storage disease X (#261670)	Muscle cramps, exercise intolerance, elevated serum creatine phosphokinase, myoglobinuria	–
*RP1L1*	Retinitis pigmentosa 88 (#618826)	Decreased visual acuity	–

OMIM disease: autosomal recessive diseases associated with variants in the selected genes; MIM reference numbers are detailed in brackets. Expected phenotypical features: main clinical features associated with each specific syndrome. Detected phenotypical features: identified HKOs clinical presentations.

### HKOs subjects presenting with the expected phenotype

#### *C7* gene

Biallelic LoF variants in this gene have been associated with C7 deficiency, a rare immunological defect characterized by increased susceptibility to systemic infections, mainly caused by encapsulated bacteria [17]. Individual_1, carrying the known NM_000587.2:c.2350+2T>C splicing variant [18], presented with the typical clinical features of C7 deficiency. Specifically, the patient suffered from a meningococcal meningitis episode and reported a long history of gastritis related to *Helicobacter pylori* infection, pericarditis, pneumonia, bronchopneumonia, and a peculiar soft tissue infection of the tip of the nose. Despite the presence of clear signs and symptoms, the disease was never diagnosed, and a genetic test was never requested by the physicians who took care of this patient.

#### *F12* gene

Biallelic LoF variants in this gene may cause Factor XII deficiency, which is usually not associated with any clinical symptom, but causes prolonged whole-blood clotting time [19]. All the LoF variants in this gene described in literature are associated with Factor XII deficiency, except for a small insertion and a gross deletion, causative of hereditary angioedema [20]. Here, the NM_000505.3:c.1681-1G>A splicing variant was detected in Individual_2 at the homozygous state. Blood coagulation tests were not performed during the initial evaluation and no other peculiarities emerged from the patient’s clinical assessment, but during the follow-up visit, the subject reported a history of extended coagulation time with an activated partial thromboplastin time of 200–300 s (average values: 30–40 s). Despite the altered coagulation time, Factor XII deficiency was never suspected, and a genetic test was never performed.

#### *GPR68* gene

Biallelic LoF variants in this gene have been associated with Amelogenesis imperfecta type IIA6, characterized by enamel hypomineralization, which causes early functional failure [21]. Individual_3 is a homozygous carrier of the NM_003485.3:c.1006G>T nonsense variant in the *GPR68* gene, and at follow-up reported a history of multiple caries and recurrent tooth decay since childhood; the subject has been wearing dentures since the age of 20 years. Also, in this case, despite the presence of clinical features characteristic of Amelogenesis imperfecta, the subject was still lacking the precise clinical diagnosis and subsequently had never undergone genetic testing.

### HKOs subjects not presenting with the expected phenotype

#### *ACADSB* gene

Biallelic LoF variants in the *ACADSB* gene cause 2-methylbutyrylglycinuria, a metabolic disorder characterized by impaired isoleucine degradation. This disorder may be detected via newborn screening; it is often clinically asymptomatic, but some individuals have been reported to be affected by developmental delay and neurological signs and symptoms including hypotonia and seizure [22]. A homozygous splicing variant, NM_001609.3:c.303+1G>A, has been detected in Individual_4 and Individual_5, two sisters from our cohorts. This variant has not previously been associated with 2-methylbutyrylglycinuria; another nucleotide change involving the same splicing site (NC_000010.10:g.124797366A>G) has been described as causative of this disease [23]. None of the two subjects presented with neurological alteration during our assessment nor reported developmental difficulties during childhood.

#### *FANCL* gene

**Fig. 3:**
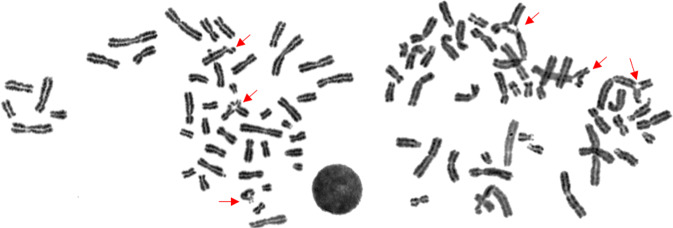
Diepoxybutane (DEB) chromosome fragility test. The figure shows the outcome of the DEB test performed on the carrier of the NM_018062.3:c.2T>C variant in the *FANCL* gene. Red arrows indicate the typical triradial and quadriradial chromosome breakage patterns.

Biallelic LoF variants in this gene have been associated with Fanconi anemia (FA), a severe condition usually lethal in childhood [24]. In Individual_6, we detected a rare biallelic LoF variant, NM_018062.3:c.2T>C, which has been described as causative of breast cancer in males [25]. At the initial clinical evaluation, the woman reported a history of head and neck carcinoma and short stature, both possible signs of FA. At follow-up, the diepoxybutane (DEB) chromosome fragility test, pathognomonic of FA [26], resulted positive, as shown in Fig. 3, even though no classical hematological FA pattern was found in the subject both at first sampling and at follow-up (white blood cell count: 5.16 × 10^3^/μl (normal values: 3.7–11.7 × 10^3^/μl); red blood cell count: 4.67 × 10^6^/μl (normal values: 3.88–5.78 × 10^6^/μl); platelets: 277 × 10^3^/μl (normal values: 172–400 × 10^3^/μl)). Moreover, three relatives of Individual_6 were also investigated (her two children, a 48-year-old man and his sister of 51 years of age, and her brother, a 71-year-old man). They are carriers of the variant at the heterozygous state, and, as expected, none of them presented any peculiar phenotype nor a positive DEB test.

#### *GRK1* gene

The majority of the biallelic LoF variants in this gene are responsible for Oguchi disease type 2, a congenital stationary night blindness in which every other visual function—visual acuity, visual field, and color vision—are usually normal [27]. Moreover, one of the nonsense variants and one of the small insertion previously described have been linked to autosomal recessive retinal dystrophy [28] and retinitis pigmentosa [29], respectively. In Individual_7, we identified the NM_002929.2:c.699+1G>A splicing variant at the homozygous state. The analysis of the clinical and instrumental data carried out during the initial assessment on the subject excludes the presence of retinal disease or any other signs of Oguchi disease type 2 since he did not specifically report any visual alteration in dark adaptation. Unfortunately, no recent clinical data are available since the subject died and it has not been possible to perform a follow-up visit.

#### *LGI4* gene

**Fig. 4:**
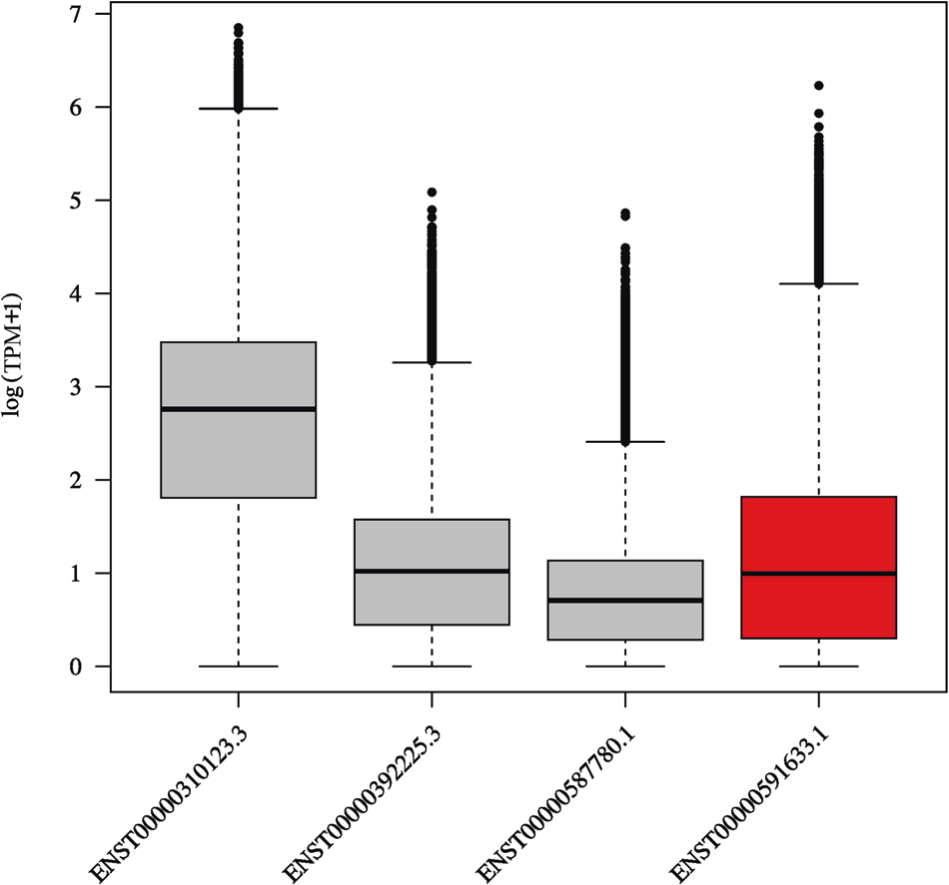
*LGI4* expression, protein-coding transcripts. The figure displays on the *y* axis the logarithmic value of the *LGI4* gene expression for protein-coding transcripts, measured in transcripts per millions (TPM), and on the *x* axis the four *LGI4* protein-coding transcripts. The red box represents the transcript of interest (ENST00000591633.1).

Biallelic *LGI4* LoF variants may cause a rare form of neurogenic Arthrogryposis multiplex congenita due to a specific myelin defect, a severe disease characterized by prenatal onset (reduced fetal mobility, club feet, camptodactyly), which often results in stillbirth. Live-born children present multiple joint contractures and usually die within a few days of respiratory failure secondary to pulmonary hypoplasia [30]. The investigated HKO, Individual_8, did not present any clinical features associated with this specific disease, reporting only hypertension and dying at 81. Further investigations of the detected ENST00000591633.1:c.636del variant highlighted that it does not impact the canonical *LGI4* transcript, and it involves only one protein-coding transcript out of the four reported in the Ensembl database. This transcript is the one with the shortest protein product, and its median mRNA expression, assessed using RNA-seq data from the Genotype-Tissue Expression (GTEx) project [31], is very low compared to the one of the canonical transcript (1.7 transcripts per million vs. 14.8 transcripts per million) (Fig. 4). All four LoF variants already described in the literature as causative of Arthrogryposis multiplex congenita fall outside our transcript of interest. Moreover, among the remaining five missense variants identified, only one (i.e., NM_139284.2:c.200A>C) affects our transcript and, to our knowledge, no disease-causing variants specifically affecting the coding region of this isoform have ever been described.

#### *MPO* gene

Biallelic LoF variants in this gene have been associated with Myeloperoxidase deficiency, a primary immunodeficiency due to a defect in innate immunity, which may lead to an increased incidence of fungal infection, particularly candidiasis [32]. Individual_9, who carries the known NM_000250.1:c.1552_1565del variant [33], is an *MPO* HKO who did not report episodes of recurrent candidiasis or other severe infections, neither at the first clinical assessment nor at follow-up.

#### *PGAM2* gene

Biallelic LoF variants in the *PGAM2* gene may be responsible for muscle phosphoglycerate mutase deficiency, known as well as glycogen storage disease X [34], or for rhabdomyolysis [35]. Affected individuals may complain of exercise intolerance, intense exertion pain, and muscle cramps; they may also present with elevated serum creatine phosphokinase (CPK) and occasional myoglobinuria [36]. In our cohorts, Individual_10 carries the NM_000290.3:c.532del variant, previously associated with muscle phosphoglycerate mutase deficiency [37]. The *PGAM2* KO subject’s blood tests only showed increased lactate dehydrogenase values (429UI/l (normal values: 140–280 UI/l)) with CPK within the normal range (165 UI/l (normal values: 24–204 UI/l)); anamnestic and clinical data did not suggest exercise intolerance or exertion pain.

#### *RP1L1* gene

Specific biallelic LoF variants in this gene may cause autosomal recessive Retinitis pigmentosa; moreover, other variants in this gene may cause occult macular dystrophy. Symptoms of patients carrying LoF variants usually include night blindness, tunnel vision, slowly progressive decreased central vision, decreased visual acuity, visual field alteration, dyschromatopsia, and alterations at the fundus oculi examination [38]. Here, we identified a HKO (Individual_11), carrying the NM_178857.5:c.326_327insT variant, associated with syndromic retinal dystrophy in a previously described patient carrying another *in cis RP1L1* nonsense variant (NM_178857.5:c.326_327insA) together with a nonsense variant in *C2orf71*, thus suggesting a digenic effect [39]. Our subject did not report any history of ophthalmologic disorders.

## Discussion

One of the major goals in biomedicine consists in understanding the function of every gene of the human genome. An interesting approach to achieve this is represented by the study of putative LoF variants that disrupt both copies of a specific gene. A key point in studying HKOs consists in the identification of populations that may be enriched in these rare and possibly disease-causing LoF variants, such as genetic isolates. Only a few research groups have so far focused on this specific kind of population. For example, Saleheen et al. [40]. have recently described a series of Pakistani adult HKOs detected during a study aimed at identifying variants influencing cardiovascular disease. In 2015, more than 1100 homozygous LoF variants were detected in a cohort of over 100,000 Icelanders [41], and the following year over 780 HKOs have been identified in a cohort of consanguineous British adults [42]. One overall advantage of this kind of study is the possibility to perform in-depth phenotyping with accurate follow-up to link the identified LoF variants to a specific clinical outcome [4].

In this study, we describe the results of the first Italian screening of HKOs by combining WGS data and deep phenotyping. This work represents a further detailed characterization of the initial analysis of knockout variants carried out by Cocca et al. [12]. In particular, we focused on LoF variants involving OMIM disease-associated genes, and specifically on those linked to autosomal recessive disorders, in order to be able to objectively assess whether the identified variants were associated with a well-known clinical condition. Our results may be summarized in two classes: (1) HKOs presenting the expected phenotype, in most cases not diagnosed, and (2) HKOs that, despite carrying biallelic homozygous LoF variants, do not display the supposed clinical outcome.

The carriers of biallelic LoF variants in three genes, *C7*, *F12*, and *GPR68*, belong to the first group. As regards *C7* deficiency, the investigated subject reported the typically increased infection rate. Despite the clear clinical signs, a genetic condition was never suspected, thus not allowing the patient to benefit from preventive medical strategies such as meningococcal vaccination or plasma transfusion. The *F12* gene KO individual presented a history of extended coagulation time without any other relevant clinical problem. In this case, as well, no genetic condition was suspected, and the patient’s surgical procedures were repeatedly delayed because of the impossibility to understand the reason of the coagulation defect correctly. Furthermore, a *GPR68* HKO reported Amelogenesis imperfecta distinctive clinical manifestations. Again, this individual never received a genetic disease diagnosis, which could have led to an early therapy based on enamel protection and specific dental surgery.

In the second category, for four HKOs (*ACADSB*, *MPO*, and *PGAM2* genes, respectively) the expected clinical phenotype was not detected. According to literature data, only 10% of the subjects carrying biallelic LoF variants in the *ACADSB* gene develop early childhood symptoms, especially when exposed to increased catabolic stress, which may lead to metabolic decompensation [43]. Similarly, *MPO* HKOs are usually asymptomatic, not displaying an increased susceptibility to infections, unless specific comorbidities occur (i.e., diabetes mellitus) [44]. Regarding *PGAM2*, LoF variants carriers become symptomatic only during strenuous physical exercise and are otherwise asymptomatic [45]. Therefore, the absence of clinical signs and symptoms in these four HKOs may be due to the specific incomplete penetrance of the underlying diseases. In this category, other interesting results are represented by the discovery of two different subjects carrying biallelic LoF variants in the *GRK1* and *RP1L1* genes, both involved in retinal diseases. The detected KO carriers did not report a history of ophthalmologic disorder or visual alteration in dark adaptation. Again, in this case, literature data suggest that both conditions are mild and non-progressive. In this light, since these pathologies peculiar clinical signs might have been missed, it would be proper to perform a deep and updated ophthalmological evaluation on the *RP1L1* HKO (i.e., fundus oculi assessment). We additionally identified an *LGI4* HKO who did not present the expected clinical features. The finding was striking since biallelic LoF variants in this gene cause a severe disease that often results in stillbirth or neonatal death. However, meticulous analysis of the variant genomic context showed that it does not impact the canonical *LGI4* transcript, which still seems able to generate the full-length protein, thus explaining the typical clinical phenotype absence in the detected HKO. Finally, the most intriguing case is represented by discovering an HKO for the *FANCL* gene, which, when mutated, causes FA, a severe genetic disease often lethal in childhood. The KO carrier we identified is a 74-year-old individual characterized by short stature who reported having suffered from a brain tumor and a head and neck carcinoma without showing the classical FA spectrum phenotype (i.e., not presenting any hematological abnormalities) but with a positive DEB test. The reasons for the mild clinical presentation of this HKO are still unclear. Several hypotheses may be proposed: (a) the possible presence of other variants in the *FANCL* gene that might allow the transcription of a shorter transcript leading to the production of a smaller but still partly functioning protein, and (b) the possibility that this individual carries other variants/genes able to compensate for the detrimental effects of the disease-related *FANCL* allele. Future in vitro and in vivo studies will clarify if this “genetic resilience” is related to a secondary variant that bypassed the mutant phenotype or a gene over-expression that rescued the mutant phenotype.

In conclusion, the present findings remark the importance of a deep phenotypical characterization when trying to understand the role of LoF variants, performing, when required, a specific clinical follow-up on all HKOs. The detection of KO subjects presenting the expected phenotype highlights how often the lack of a correct diagnosis, including a genetic one, may lead to inappropriate or delayed treatment. On the other hand, the identification of subjects that, despite carrying biallelic LoF, do not display a conventional clinical presentation, underlines how LoF variants may be responsible for a broader phenotypic spectrum than previously expected, raising awareness toward the discovery of putatively protective variants that may become the cornerstone of new therapeutic approaches. Overall, studying HKOs in genetic isolates represents an intriguing and not commonly employed opportunity to investigate genotype–phenotype correlations, with still undiscovered potential in helping the clinical decision-making process regarding preventive, diagnostic, and therapeutic approaches.

## Supplementary information

Supplementary Table 1
